# Botanical Report

**Published:** 1812-03

**Authors:** 


### BOTANICAL REPORT.

SINCE our last Report, two Numbers of the BOTANICAL MAGAZINE have been submitted to our inspection, the contents of which we shall here briefly comment upon.

ZINGIBER *casumunar*, of Roxburgh, who, in the eleventh volume of the Asiatic Researches, has favored the botanical world with an account of the plants belonging to the natural order of *scitamineæ*, which have fallen under his observation in the East Indies. Situated at the botanic garden in Calcutta, this author has had excellent opportunities of studying these curious vegetables, and has not failed to make proper use of the opportunities afforded him. The specimen from which the drawing was taken, flowered, probably for the first time in Europe, in Mr. Vere's stove at Kennington-Gore, in August last. The root of this plant was some year's ago introduced as a medicine of uncommon efficacy in several nervous disorders, but is not now to be found in our shops. This plate contains a diminished outline representation of the whole plant, which adds much to its value. As this species was not known to Mr. Roscoe, when he wrote his excellent essay on the natural order of Scitamineæ in the eighth volume of the Transactions of the Linnæan Society, it is pleasing to observe that the generic character taken from the filament corresponds with the rest, and thus confirms the propriety of the genus, as established by this learned botanist.

GLOBBA *sessiliflora*, although not the next in rotation, is another plant plant of the same natural order, from the collection of Sir Abraham Hume, and is probably the G. *bulbifera* of Roxburgh; as is the more probable from its being received by Sir Abraham from this author, though, from the scanty account of the last-mentioned species in the Asiatic Researches, Dr. Sims could not decide it with sufficient certainty to adopt his name. The opportunity is taken to point out the generic difference between Globba and Mantisia.

Eryngium *corniculatum*. Singular, from having usually one of the rigid leaflets of the involucre perforating the very centre of the head of flowers. We call this remarkable beak part of the involucre, from its similarity of figure with the other leaflets, and its being sometimes wanting, at other times split into two, or even several. It is remarked from Brotero that E. *corniculatum*, *galioides*, and *odoratum*, of Lamank, are probably merely varieties of the same plant. Native of Portugal, which we mention because Mr. Donn, by whom this plant was communicated to Dr. Sims, has marked the habitat as uncertain in the last edition of his catalogue of the Cambridge garden.

Salvia *hablitziana*, a very fine shrubby species of sage, introduced by Dr. Clarke from the valley of Tchorgana in Tauria, and communicated by Mr. Donn from the botanic garden at Cambridge.

Amaryllis *purpurea*. Linnæus compared the natural order of lilies to the nobles of the land, adorning the nation by the gaudy richness of their attire, and the spendor of their equipages. Of the genus Amaryllis, one of the first of the patrician families, the present species is perhaps the most superb. The drawing was made at Messrs. Middlehurst and Wood's nursery, at Shepherd's-bush.

Mr. Ker has taken the opportunity of giving more correct characters of some of the species of this genus which have been before published in the Magazine. Am. *humilis* (β) he is now convinced is distinct, and has given it the name of *corusca*, *undulata* and *venusta* have new specific characters: the last is acknowledged to be hardly distinct from *sarniensis*, of which it is made a variety in the Hortus Kewensis.

Commelina *africana*, with a new generic character from Brown's prodromus; and an observation from the late Mr. Dryander, that the Commelina *Africana* of Redouté (Liliac t. 207), is certainly a different species.

To this article Mr. Ker has added a note on the Sanseviera *sessiliflora*, correcting an error he had fallen into, in supposing that its scape was extrafoliaceous. He has added, as synonyms, S. *carnea*, Bot. Rep. Lil. a Redouté and Hort. Kew. ed. 2.

Allium *paniculatum*. Mr. Ker has taken great pains to unravel the confused synonymy of this genus, which, however, in some cases, seems to be quite impossible. He has omitted quoting, in this instance, the *paniculatum* of most of the more recent European Floras, from a suspicion the plant they mean is generally a capsuliferous variety of *oleraceum*. Mr. Ker remarks, it is very possible that some of the authors cited under *pallens* (No. 1420 of the Botanical Magazine), had the plant now given as *paniculatum* in their

 minds, minds, and that the above said *pallens* is in reality a capsuliferous variety of *carinatum*. *Oleraceum* and *carinatum*, indeed, occurring without bulbs, Mr. Ker is persuaded, have afforded *pallens* and *paniculatum* to most of the modern Floras; we understand that he means, by the above account, to exclude *pallens* altogether as a distinct species.

HEMEROCALLIS *japonica*. The white sweet-scented day-lily of Japan; quite a distinct species from the blue Japan day-lily. It is observed here that it blooms later and not so freely as the blue sort. We add that the latter succeeds perfectly well when treated as a hardy herbaceous plant, but the white sort succeeds best in the greenhouse.

SABAL *Adansoni;* the American Swamp Palmetto. M. Guersent, in the Bulletin de la Societé Philomat. has observed that this plant cannot belong to Raphis, Chamærops, or Corypha, to all of which it has been referred, nor to Euterpe, on which account he has retained Adamson's barbarous name of Sabal. Mr. Ker, pleading his little acquaintance with these plants, has implicitly followed Guersent, without pretending to judge of the solidity of his generic distinctions.

TRADESCANTIA *cristata*. Mr. Brown thinks this plant ought to be separated from Tradescantia. There is a singularity of appearance, but not much beauty, in this neglected annual, which we have several times known to intrude itself uninvited, coming up spontaneously in the mould sent with other plants from the East Indies.

COTYLEDON *crenata;* VEREA of Wildenow, and Calanchoe of Adanzon and Persoon. Dr. Sims, having before adopted the Bryophyllum of Salisbury, upon nearly the same grounds as this has been separated from Cotyledon, was probably induced to forego adopting the genus Calanchöe, out of deference to the new edition of Hortus Kewensis.

CYMBIDIUM *coccineum*. One of the parasitical *orchideæ*, which were thought by Philip Miller not to be capable of artificial cultivation in our northern climate. But more modern gardeners have succeeded with many of them admirably well, by planting them in mould consisting chiefly of decayed wood, and covering the surface with large pieces of tanners' bark. The present specimen came from Messrs. Loddiges.

CYTISUS *bucanthus*. Native of Hungary, first published in the Plantæ rariores of that country. Cultivated in the botanic garden at Cambridge, and communicated from Mr. Donn.

ALSTROGOMA *humifurum*. VINTENATIA of Cavanilles, native of New Holland. A genus established by Mr. Brown in his Prodromus; but appears to be very nearly allied to Styphelia.

In the course of last month, we have seen the 1st number of a new botanical work, intitled "SKETCHES towards a HORTUS."

BOTANICUS AMERICANUS, with colored plates. By William Thetford, M.D. and corresponding member of the Society for the Encouragement of Arts, &c. a great curiosity certainly in its way, and and of which it is not very easy to communicate a just idea to our readers. We will attempt it however: Imagine, then, some good mamma, with her dear little boy upon her knee, and her pencil in her hand. Now, "Pray mamma draw me the sugar-cane:"—"There it is my dear!"—"Now draw me a cashew nut:"—"There it is!"—"Now a tamarind-tree:"—"See, there it is!"—"A pine-apple, mamma:"—"There dear!"—"Now, dear mamma, do draw me a coffee-tree:"—"A coffee-tree, Jackey, bless me, I forget:"—"Oh, don't you remember, it grew under the parlor window, and Ben used to train its branches so pretty along the wall?"—"Oh, yes; now I have it—there it is, with its pretty red berries." Next you must suppose, that little Master Jackey was a very good boy, and took great care of all the little drawings mamma used to make him. Then comes papa, and unluckily takes it into his head that these nice little figures, with the botanical names added to them, would make a very pretty publication. So he sets himself to work, arranges them according to the sexual system, in rows, one below another, some large and some small, just like the penny pictures we every day see little masters and misses cutting to pieces in our nurseries; except, indeed, that these latter are rather less unlike the things they are meant to represent. Such figures, though, with some affectation of modesty, announced as sketches only, we did not expect to see in the nineteenth century!

We ought, however, to mention, that these miniature pictures appear to be only introductory; and that the two last plates contain drawings as large as life; but even these are not much better.

It is a remark of some writer, that there is no book so bad but something may be learned from it; and we were really in hopes of being informed what plant the Jamaica wild-clary was, said to be in common use there as a domestic remedy; but we have not been able to detect it from the figure here given; nor can we receive any assistance from our author, who refers it to Salvia *sclarea,* the clary of Europe, to which it certainly possesses no affinity, nor even resemblance; and must have taken its name, if such name it really bear, from some similarity of smell or taste, or medicinal efficacy, certainly not from any agreement in their external characters. If the future Numbers should afford us any useful information, we shall take further notice of this work at a future time.

